# Cytomegalovirus, Epstein-Barr virus and human herpesvirus 8 salivary shedding in HIV positive men who have sex with men with controlled and uncontrolled plasma HIV viremia: a 24-month longitudinal study

**DOI:** 10.1186/s12879-018-3591-x

**Published:** 2018-12-19

**Authors:** Monica Basso, Samantha Andreis, Renzo Scaggiante, Elisa Franchin, Daniela Zago, Maria Angela Biasolo, Claudia Del Vecchio, Carlo Mengoli, Loredana Sarmati, Massimo Andreoni, Giorgio Palù, Saverio Giuseppe Parisi

**Affiliations:** 1 Department of Molecular Medicine, University of Padova, Via Gabelli 63, 35100 Padova, Italy; 2 Infectious Diseases Unit, Padova Hospital, Via Giustiniani 2, 35128 Padova, Italy; 3 Clinical Infectious Diseases, Tor Vergata University, viale Oxford, 81, 00133 Rome, Italy

**Keywords:** HIV positive MSM, Longitudinal persistence of salivary shedding, EBV DNA, HHV-8 DNA, CMV DNA

## Abstract

**Background:**

This longitudinal study described Cytomegalovirus (CMV) DNA, Epstein-Barr (EBV) DNA and human herpesvirus 8 (HHV-8) DNA asymptomatic salivary shedding in HIV-positive men who have sex with men (MSM). We aimed to 1-analyze frequency and persistence of herpesvirus shedding, 2-correlate herpesvirus positivity and HIV viroimmunological parameters and 3-assess the association between HIV-RNA suppression and herpesvirus replication.

**Methods:**

Herpesvirus DNA was tested with an in-house real-time PCR in 2 salivary samples obtained at T0 and T1 (24 months after T0). HIV-RNA was evaluated in the 24 months prior to T0 and in the 24 months prior to T1; MSM were classified as successfully suppressed patients (SSPs), viremic patients (VPs) and partially suppressed patients (PSPs). EBV DNA load was classified as low viral load (EBV-LVL, value ≤10,000 copies/ml) and as high viral load (EBV-HVL,> 10,000 copies/ml). Mann-Whitney U test tested the difference of the median between groups of patients. Chi-squared test and Fisher’s exact test compared categorical variables according to the frequencies. Kruskal-Wallis test compared continuous data distributions between levels of categorical variables.

**Results:**

Ninety-two patients (median CD4+ count 575 cells/mm3, median nadir 330 CD4+ cells/mm3) were included: 40 SSPs,33 VPs and 19 PSPs. The more frequently single virus detected was EBV, both at T0 and at T1 (in 67.5 and 70% of SSPs, in 84.8 and 81.8% of VPs and in 68.4 and 73.7% of SPSs) and the most frequently multiple positivity detected was EBV + HHV-8. At T1, the percentage of CMV positivity was higher in VPs than in SSPs (36.4% vs 5%, *p* < 0.001), the combined shedding of HHV-8, CMV and EBV was present only in VPs (15.1%, *p* = 0.01 respect to SSPs) and no VPs confirmed the absence of shedding found at T0 (vs 17.5% of SSPs, *p* = 0.01). EBV-HVL was more frequent in VPs than in SSPs: 78.6% at T0 (*p* = 0.03) and 88.9% at T1 (*p* = 0.01).

**Conclusions:**

The relationship between uncontrolled plasma HIV viremia and CMV, EBV, and HHV-8 shedding is multifaceted, as demonstrated by the focused association with EBV DNA load and not with its frequency and by the persistent combined detection of two oncogenic viruses as EBV and HHV-8 regardless of HIV virological control.

**Electronic supplementary material:**

The online version of this article (10.1186/s12879-018-3591-x) contains supplementary material, which is available to authorized users.

## Background

Adult humans harbour a variable number of persistent viruses, most of which do not cause disease in healthy people [1]. However, the reactivation of members of the herpes-virus family such as Epstein-Barr virus (EBV), Cytomegalovirus (CMV) and human herpesvirus 8 (HHV-8) may be responsible for serious diseases in the infected host [2]. CMV, EBV and HHV-8 are DNA viruses that can infect a broad spectrum of cellular types. CMV primarily infects monocytes and epithelial and vascular endothelial cells [3, 4], whereas the main targets for EBV infection are B lymphocytes and epithelial cells [5] and HHV-8 cellular tropism include endothelial cells, monocytes and B cells [6].

EBV infection is associated with cancers such as Burkitt’s lymphoma, Hodgkin’s lymphoma, a subset of gastric carcinomas, and almost all undifferentiated nasopharyngeal carcinomas [7]; an oncomodulatory role for CMV was proposed in high-grade glioblastomas [8], whereas Kaposi sarcoma, multicentric Castleman disease, and primary effusion lymphoma are associated with HHV-8 infection [9]. Interestingly, these three herpesviruses are also involved in inflammatory disorders, such as HHV-8-inflammatory cytokine syndrome [10]; additionally, CMV and EBV replication may be linked to the development of autoimmune diseases including systemic lupus erythaematous and systemic sclerosis [11, 12].

Saliva collection is simple and non-invasive. The detection of herpesvirus DNA in saliva samples has a potential diagnostic role: testing CMV PCR in saliva can be included in the workflow to diagnose congenital CMV infection [13]. For example, EBV DNA was detected in 80% of pretreatment saliva samples in 175 consecutive newly diagnosed nasopharyngeal carcinomas cases [14], and HHV-8 oral shedding was positively related to HPV infection in the anal area in HIV-positive men who have sex with men (MSM) [15]. Only a few studies have evaluated CMV DNA, EBV DNA and HHV-8 DNA simultaneously in salivary samples of HIV patients: one study had a cross-sectional design [16], one was a case-control study [17], one reported a comparative analysis between the results obtained in HIV-positive subjects and age- and sex-matched HIV seronegative controls [18], and Jacobson et al. [19] performed a small pilot study that analysed 2–4 saliva specimens obtained from a prospective observational study spanning a 6–12 month period. In our previous study [20], which included 193 HIV-positive MSM, we showed that 76.2% of the entire cohort displayed detectable EBV DNA in their saliva. Additionally, CMV DNA was found in 18.7% of the subjects, and a double herpesvirus positivity was reported in 16.6% of the patients. Isolated EBV shedding was reported in a comparable percentage of patients with successful plasma HIV viremia suppression and in those with not suppressed viremia (60 and 59.7%, respectively), whereas the combined shedding of the two herpesviruses was more frequent in patients with uncontrolled plasma HIV RNA (25% versus 11.8%, respectively).

This longitudinal study described CMV DNA, EBV DNA and HHV-8 DNA asymptomatic salivary shedding in HIV-positive men who have sex with men (MSM). We had three main objectives:1-to analyze the frequency and persistence of single and combined herpesvirus shedding; 2- to evaluate the correlations between herpesvirus positivity and viroimmunological parameters of HIV disease (CD4+ and CD8+ lymphocyte count and percentage, CD4/CD8 ratio, and CD4+ count at nadir); 3-to determine if long-term plasma HIV viremia successful suppression correlated with herpesvirus replication control. The patients were followed for a 24-month study period before the first saliva sampling and for a 24-month interval before the second saliva sampling.

## Methods

HIV-positive MSM older than 18 years of age were enrolled from January 2013 to December 2013. The patients had to fulfil the following inclusion criteria: 1) diagnosis of chronic HIV infection; 2) availability of regular monitoring of HIV disease (at least three plasma HIV-RNA determinations by year) in the 24 months prior to each salivary sampling; 3) serology tests indicating past EBV, past CMV and past HHV-8 infection; 4) no clinical history or serological evidence of acute CMV and/or acute EBV and/or acute HHV-8 infection in the 2 years prior to enrolment; 5) no history of clinical malignancy and no oral/mucosal lesions upon oropharyngeal examination; 6) no diagnosis of Kaposi sarcoma or of other HHV-8-associated diseases; and 7) no use of drugs with activity against herpesvirus in the 48-month study period. All participants provided written informed consent for all study procedures and for the use of their data for scientific evaluation and publication in a blinded form. This study was conducted in accordance with the Declaration of Helsinki, and it was approved by the Ethical Committee for Clinical Experimentation, Padua Province (prot. 2606-12P).

The study time defined as T0 corresponded to the first saliva sampling, and the study time defined as T1 corresponded to the second saliva sampling; plasma HIV-RNA was analysed for a period of 24 months prior to T0 and for a period of 24 months prior to T1.

Viroimmunological data collected at the time of the first salivary sampling for analysis included CD4+ lymphocyte count (cells/mm^3^) and percentage, CD8+ lymphocyte count (cells/mm^3^) and percentage, CD4/CD8 ratio, and CD4+ lymphocyte count (cells/mm^3^) at nadir.

The plasma HIV RNA value was analysed separately for each of the two 24-month periods before the first and second salivary testing. Patients were considered successfully suppressed patients (SSPs) if they had undetectable plasma HIV viremia or an HIV RNA value < 400 copies/ml in all the tests performed [21]; otherwise, the subjects were classified as viremic (viremic patients, VPs). The cut-off of 400 copies/ml was chosen in order to classify the subjects with a viral blip as VPs because a blip magnitude of > 400 copies HIV-1 RNA/mL may predict HIV virological failure [22, 23]. Subjects who switched from successful to unsuccessful control of plasma HIV viremia and vice versa were classified as partially suppressed patients (PSPs).

The patients provided an unstimulated saliva sample (2 ml) according to the method previously described by Navazesh [24]. The sample was kept at 4 °C, submitted within 2 h from collection to the Laboratory of Virology at the University of Padova and stored at − 80 °C until analysis.

### Laboratory methods

DNA extraction and purification of the saliva samples (600 μl, processed as plasma samples) were performed using the QIAamp Blood kit (Qiagen, Inc., Chatsworth, CA, USA).

A quantitative real-time PCR detection of EBV DNA, CMV DNA and HHV8 DNA, was performed on an ABI PRISM 7900 HT Sequence Detection System (Applied Biosystems, Foster City, CA, USA) using Taqman® technologies [25].

Primers and probe (contains a fluorescent reporter dye (6-carboxyfluorescein, FAM) at the 5′-end and a fluorescent quencher dye (6-carboxy-tetramethyl-rhodamine, TAMRA) at the 3′-end), selected using Primer-Express software, are reported:

- EBV amplification: fw 5’EBT (5’-TCA ACC TCT TCC ATG TCA CTG AGA-3′), rev 3’EBT (5′-TGG GTG AGC GGA GGT TAG TAA-3′) and probe EBpr (5’-TCA GCC CCTCCA CCA GTG ACA ATTC-3′); [26]
- CMV amplification: fw CMT-5 T (5’TCATCCACACTAGGAGAGCAGACT 3′), rev CM-3 T (5’GCCAAGCGGCCTCTGAT), and probe (5’ACTGGGCAAAGACCTTCATGCAGATCTC3’) [27];
- HHV-8 amplification: fw (5’-CTCGAATCCA ACGGATTTGAC-3′), rev (5’-TGCTGCAGAATAGCGTGCC-3′) and probe (5’-CCATGGTCGTGCCGCAGCA-3′) [28].

The human genomic β-globin DNA amplification, was performed for each sample with the same amount of extracted DNA and identical thermal cycling conditions, assessed the absence of PCR inhibitors as well.

During all DNA extractions and purifications, precautions were taken to reduce the risk of false-positive results. EBV DNA and CMV DNA viral loads were expressed in copies/mL with a threshold level of 1000 copies/ml whereas HHV-8 DNA was analysed as a binary variable (present versus absent) because no validated method to quantify this herpesvirus was available.

### Statistical analysis

The continuous data were presented as the median and interquartile range for continuous variables (years, CD4 positive cell count and percentage, CD8 positive cell count and percentage, CD4/CD8 ratio, CD4 positive count at nadir, plasma HIV-RNA value) because the data distribution was not normal: the Mann-Whitney U test was used to test the significance of the difference of the median between the groups of patients. Each specific herpesvirus shedding was analyzed as a categorical variable (i.e. CMV, EBV and HHV-8 present versus absent): all possible combinations were evaluated, including the absence of herpesvirus shedding and the simultaneous presence of the three herpesvirus. EBV-DNA shedding was also analyzed recording as variables the absolute number and proportion of patients with EBV-HVL and EBV-LVL. The Chi-squared test and Fisher’s exact test were used to compare categorical variables as appropriate (according to the frequencies). The Kruskal-Wallis test was applied to compare continuous data distributions between levels of categorical variables [29].

The limit of significance for all analyses was established at *p* < 0.05. All statistical analyses were performed with MedCalc Statistical Software version 18 (MedCalc Software bvba, Ostend, Belgium; www.medcalc.org; 2018).

## Results

### Characteristics of the subjects

Ninety-two patients (all Caucasian) with a median age of 43 years (IQR 36–51 years) were included. Plasma HIV viremia was always detectable throughout the study time (48 months) in 33 subjects (35.9%) and was always suppressed in 40 subjects (43.5%); additionally, 19 patients (20.6% of all included MSM) switched from successful to uncontrolled plasma HIV RNA or vice versa. All SSPs were on HAART (highly active antiretroviral therapy), as were the PSPs with suppressed plasma HIV viremia in the 24 months before T0 (7 patients) or in the 24 months after T0 (12 patients). Overall, median number of HIV-RNA assessments was 8 (IQR 7–9), significantly higher in PSPs (9, IQR 8–9) with respect to SSPs (7, IQR 6–8) and to VPs (8, IQR 6.7–8).

Most patients (38, 64.4% of the subjects on HAART) were treated with the nucleosidic backbone associated with a boosted protease inhibitor.

The viro-immunological characteristics of the patients at T0 are reported in Table 1. The CD4 positive cell count was comparable in the three subgroups of patients. Conversely, the CD8 positive count was lower in SSPs with respect to the other two groups.

**Table 1:** Characteristics of the 92 patients at T0 according to plasma HIV viremia control (SSP, VP and PSP). Data are expressed as median and interquartile range (IQR)

	SSP (40 pts)	VP (33 pts)	SPS (19 pts)
Age (years)	47 (39–59	40 (32–47)	43 (38–49)
CD4 + cell count/mm^3^	605 (475–785)	570 (410–662)	530 (370–827)
CD4+ cell percentage	27.5 (22.5–36)	23 (19.7–30)	24 (20–28.2)
CD8+ cell count/mm	849 (602–1227)	1106 (1012–1464)	1240 (854–1490)
CD8 + cell percentage	43 (34.5–51)	51 (47–60)	48 (42–62)
CD4/CD8 ratio	0.8 (0.5–1)	0.4 (0.4–0.6)	0.6(0.3–0.7)
CD4+ cell count at nadir (cells/mm^3^)	290 (132–405)	460 (322–535)	260 (88–355)
Plasma HIV-RNA value in the 24 month prior T0 log (Log_10_ copies/ml)	–	4.57(3.99–4.8)	–
Plasma HIV-RNA value in the 24 month prior T1 (Log_10_ copies/ml)	–	4.44 (3.77–4.83)	–

T0: time of the first saliva sampling after 24 months of plasma HIV RNA control analysis

T1: time of the second saliva testing, 24 months after T0

*SSPs* HIV successfully suppressed patients, *VPs* HIV viremic patients, *PSPs* HIV partially suppressed patients

Based on the results obtained, EBV DNA was quantified as follows: low viral load (EBV-LVL, value ≤10,000 copies/ml) and high viral load (EBV-HVL, > 10,000 copies/ml). All CMV DNA values were lower than 10,000 copies/ml and for this reason CMV DNA detectability was analysed only as a binary variable (absent vs present).

### Overall frequency and persistence of single and combined herpesvirus shedding

Overall, CMV DNA was detected in 18 subjects (19.6%) at T0 and in 16 patients (17.4%) at T1. The frequency of EBV DNA and of HHV-8 DNA positivity was higher (73.9% at T0 and 75% at T1 for EBV and 38% at T0 and 37% at T1 for HHV-8). EBV DNA shedding was the most frequently detected positivity at both T0 and T1 in all the three categories of patients. The double detection EBV-HHV-8 and the triple detection EBV, HHV-8 and CMV were those detected with the higher percentage both in SSPs and in VPs at T0 .At T1, the percentage of patients positive for CMV was significantly higher in HIV viremic patients than in subjects successfully suppressed (36.4% vs 5%, *p* < 0.001).

**Fig. 1:**
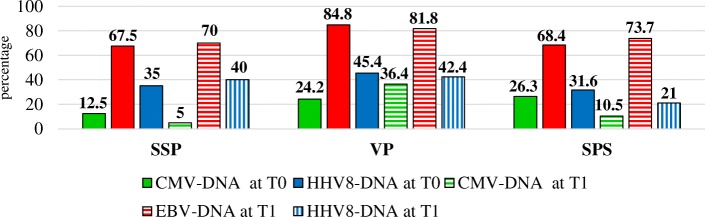
Percentage of patients with CMV DNA, EBV DNA and HHV-8 DNA salivary shedding at T0 and at T1 classified for plasma HIV RNA control. T0: time of the first saliva testing after 24 months of plasma HIV-RNA control analysis. T1: time of the second saliva testing, 24 months after T0. SSPs: HIV successfully suppressed patients. VPs: HIV viremic patients. PSPs: HIV partially suppressed patients

**Fig. 2:**
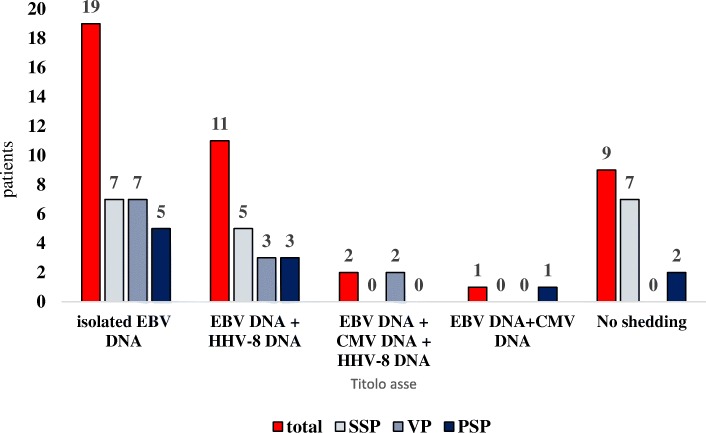
Description of the herpesviruses patterns found in salivary samples of the same patient both at T0 and at T1. Data are expressed as absolute value. *T0: time of the first saliva testing after 24 months of plasma HIV RNA control analysis. T1: time of the second saliva testing, 24 months after T0. SSPs: HIV successfully suppressed patients. VPs: HIV viremic patients. PSPs: HIV partially suppressed patients*

A complete description of absolute values of herpesvirus detection at T0 and T1 in SSPs, VPs and PSPs is presented in Fig. 1. Five herpesvirus shedding patterns were present in the patients at both T0 and T1 in the study population: isolated EBV shedding (19 patients), combined EBV-HHV-8 shedding (11 patients), combined EBV-CMV-HHV-8 shedding (2 patients), combined EBV-CMV shedding (1 patient) and no shedding (9 patients, in 17.5% of SSPs and 0 VPs, *p* = 0.01) (Fig. 2). A detailed analysis of the detectability of HHV-8, EBV and CMV at T0 and T1 is reported in Additional file 1.

### Correlations between herpesvirus positivity and viroimmunological parameters of HIV disease

At T0, VPs with HHV-8 shedding had a significantly higher CD4+ count at nadir than SSPs with HHV-8 detection in saliva. Notably, more differences were found in cases with no HHV-8 shedding: we showed lower CD4+ cell percentages and higher CD8+ cell counts and percentages in VPs with respect to SSPs (Table 2). Furthermore, we analyzed CD4+ and CD8+ cell count and percentage and CD4+ cell count at nadir in SSPs and VPs according to the detection or not of HHV8 DNA at T0 and we found that these parameters were comparable.

**Table 2:** Main viro-immunological parameters of SSP and of VP according to the detection or not of HHV-8 shedding at T0 regardless of the presence or absence of EBV and/or CMV salivary shedding. Data are expressed as median and interquartile range (IQR). In bold significant *P* values

	SSP with HHV-8 shedding (*n* = 14)	VP with HHV-8 shedding (*n* = 15)	p	SSP with no HHV-8 shedding (*n* = 26)	VP with no HHV-8 shedding (*n* = 18)	p
CD4 + cell count/mm^3^	590 (520–690)	600(447–677)	0.69	630(400–850)	535(310–620)	0.17
CD4+ cell percentage	26(24–36)	23 (21–30)	0.11	32 (22–36)	22.5 (15–28)	**0.01**
CD8+ cell count/mm	993 (522–1248)	1106 (1014–1403)	0.27	821 (619–1153)	1106 (1010–1524)	**0.009**
CD8 + cell percentage	44 (30–51)	50 (47–53)	0.09	43 (35–52)	53.5 (48–60)	***P*** **< 0.001**
CD4+ cell count at nadir (cells/mm^3^)	235 (140–340)	440 (340–567)	**0.001**	310(124–460)	470 (300–530)	0.09

T0: time of the first saliva sampling, after 24 months of plasma HIV RNA control analysis

*SSPs* HIV successfully suppressed patients, *VPs* HIV viremic patients

No significant difference in the main immunovirological parameters was found between SSPs with no salivary shedding and SSPs with the detection of any positivity at T0 and/or T1 and in patients with the same herpesvirus shedding pattern at T0 and at T1 (Table 3 and Table 4); however, HIV successfully suppressed subjects with persistently negative herpesvirus shedding had a lower median CD8+ cell count with respect to subjects who showed any herpesvirus shedding during the study period (800 cells/mm^3^, IQR 539–856 cells/mm^3^ vs 943 cells/mm^3^, IQR 610–1249 cells/mm^3^).

**Table 3:** Main viro-immunological parameters of SSP with no herpesvirus detection or with any herpesvirus shedding at T0 and T1. Data are expressed as median and interquartile range (IQR)

	Patients with persistently negative herpesvirus shedding (*n* = 7)	Patients with the detection of any positivity at T0 and/or T1 (*n* = 33)	p
CD4 + cell count/mm^3^	600 (250–802)	610 (480–782)	0.46
CD4+ cell percentage	31 (17.2–36)	27 (22.7–36)	0.87
CD8+ cell count/mm	800 (539–856)	943 (610–1249)	0.36
CD8 + cell percentage	43 (36.2–50.2)	43 (33.7–51.2)	0.95
CD4+ cell count at nadir (cells/mm^3^)	310 (87–456)	290 (135–380)	0.78

T0: time of the first saliva sampling, after 24 months of plasma HIV RNA control analysis

T1: time of the second saliva testing, 24 months after T0

*SSPs* HIV successfully suppressed patients

**Table 4:** Main viro-immunological parameters of HIV patients with the same herpesvirus shedding pattern at both T0 and T1. Data are expressed as median and interquartile range (IQR)

	Isolated EBV-DNA (*n* = 19)	EBV-DNA + HHV-8 DNA (*n* = 11)	EBV-DNA + HHV-8 DNA + CMV-DNA (*n* = 2)	EBV-DNA + CMV-DNA (*n* = 1)	No shedding (*n* = 9)
CD4 + cell count/mm^3^	560 (427–672)	630 (540–777)	670 (600–740)	410	600 (207–857)
CD4+ cell percentage	26 (21.2–34.7)	33 (25.2–36.7)	25.5 (21–30)	20	27 (13.7–34)
CD8+ cell count/mm	1000 (706–1316)	1123(527–1228)	1473 (1233–1714)	943	800 (646–1001)
CD8 + cell percentage	46 (40–52)	46 (30.5–52.7)	55 (50–60)	46	40(35.7–46.7)
CD4+ cell count at nadir (cells/mm^3^)	290(110–517	340 (212–480)	585(510–660)	410	310(25–450)

T0: time of the first saliva sampling, after 24 months of plasma HIV RNA control analysis

T1: time of the second saliva testing, 24 months after T0

### Long-term plasma HIV viremia successful suppression and herpesvirus replication control

At T0, SSPs and VPs had a comparable frequency of patients who experienced a single herpesvirus shedding (37.5 and 33.3%, respectively), but SSPs had a higher percentage of subjects with no shedding (27.5% vs 12.1%, respectively) and a lower percentage of combined herpesvirus shedding (35% vs 54.5%, respectively). At T1, the combined shedding of HHV-8, CMV and EBV was present only in HIV viremic patients (15.1%, *p* = 0.01 with respect to subjects successfully suppressed).

HIV viremic patients with CMV shedding at T1 had a higher median plasma HIV RNA value in the 24 months prior to T1 than in CMV-negative subjects (median value 4.78 log_10_ copies/ml, IQR 4.59–5.02 versus 4.34 log_10_ copies/ml, IQR 3.57–4.55 log_10_ copies/ml, *p* = 0.01); conversely, no statistically significant correlation between HHV-8 and EBV detection at T0 and T1 and plasma HIV viremia was shown.

**Fig. 3:**
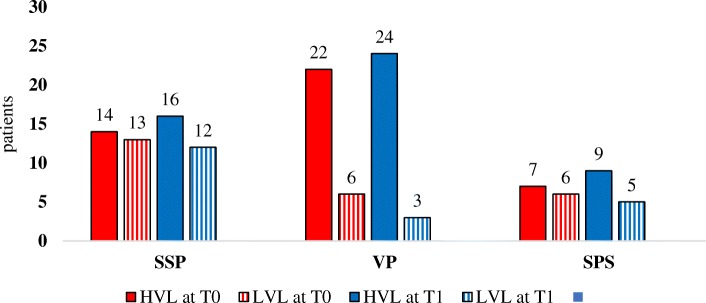
Description of positive EBV DNA results in SSPs, VPs and PSPs according to EBV DNA viral load, categorized as HVL or LVL. Data are expressed as absolute numbers. T0: time of the first saliva testing after 24 months of plasma HIV RNA control analysis. T1: time of the second saliva testing, 24 months after T0. LVL: EBV low viral load (value ≤10,000 copies/ml). HVL: EBV high viral load (value > 10,000 copies/ml). SSPs: HIV successfully suppressed patients. VPs: HIV viremic patients. PSPs: HIV partially suppressed patients

The highest percentage of patients with EBV DNA shedding who had EBV HVL was found in VPs: 78.6% at T0 (*p* = 0.03 with respect to SSPs) and 88.9% at T1 (*p* = 0.01 with respect to SSPs) (Fig. 3). Moreover, a higher frequency of VPs with no EBV shedding at T0 experienced HVL at T1 with respect to SSPs (80% vs 23.1%, respectively, *p* = 0.04).

The median plasma HIV RNA value was higher in subjects with HVL with respect to subjects with LVL at T0 (4.63 log_10_ copies/ml, IQR 4.04–4.79 copies/ml versus 3.97 log_10_ copies/ml, IQR 3.78–4.82, *p* = 0.26). At T1, only 3 subjects had LVL, so a comparative analysis could not be performed: plasma HIV viremia at in patients with EBV-HVL was 4.43 log_10_ copies/ml (IQR 3.61–4.83 log_10_ copies/ml); no significant difference with respect to plasma HIV RNA at T0.

### Key points

Overall, we showed that most HIV+ MSM had different patterns of salivary EBV DNA, CMV DNA and HHV8 DNA shedding and that ongoing plasma HIV RNA detection had an intriguing relationship with some aspects of the phenomenon. The most important results reported in patients with successful plasma HIV RNA suppression and in those with ongoing plasma HIV viremia were: 1) no HIV viremic patient was negative for herpesvirus shedding both at T0 and at T1; 2) isolated EBV DNA shedding was the most frequent figure detected both at T0 and at T1 in HIV successfully suppressed patients and in HIV viremic patients; 3) HIV viremic patients had the highest percentage of patients with HVL-EBV DNA shedding both at T0 and at T1; 4) CMV positivity was significantly more frequent in the HIV viremic patients than in HIV successfully suppressed patients at T1; 5) overall, the most frequent persistent combined shedding was EBV-HHV-8.

## Discussion

To the best of our knowledge, this is the first study designed to evaluate salivary EBV DNA, CMV DNA and HHV-8 DNA shedding in a cohort of Caucasian HIV-positive MSM that included both subjects on ART and untreated patients who were followed for 48 months and had two saliva samples obtained at a 24-month interval from one another. The inclusion of untreated patients was possible because HIV guidelines at the time of sampling [30] did not recommend the initiation of ART irrespective of the CD4+ cell count, which contrasts with updated prescriptions [31]; currently, the inclusion of untreated subject in a study lasting 48 months would be unethical.

Our observation period was the same length for all the subjects included, unlike that of Jacobson et al. [19]. These authors analysed the replication of CMV, EBV and HHV-8 in paired stored saliva specimens obtained from at least two visits separated by an interval of at least 6 months; each patient had 2–4 samples tested. Furthermore, Jacobson et al. [19] included only 16 subjects who were all on effective antiretroviral therapy for at least 1 year (plasma HIV RNA < 75 copies/mL). Overall, 27% of the saliva samples were positive for CMV, 73% were positive for EBV and 24% were positive for HHV-8. The comparison of the proportion of herpesvirus detection frequencies described in Jacobson’s work [19] and in SSPs should take into account the different number of samples collected for each patient (2–4 samples versus one at each study time). However, the percentages of EBV detection reported by Jacobson et al. [19] were comparable to those we reported (67.5% at T0 and 70% at T1), whereas the frequency of CMV replication was higher than that of ours (12.5% at T0 and 5% at T1), and the frequency of HHV-8 was lower (35% at T0 and 40% at T1). We reported a CMV positivity rate at T0 similar to the 10% found by Dittmer et al. [16] in HIV patients who had more than 200 CD4+ cells/mm^3^, regardless of the plasma HIV RNA value (evaluated once). Interestingly, we observed a lower CMV DNA positivity value at T1 (5%), after an additional 24 months of successful plasma HIV RNA control, suggesting a relationship between HIV RNA and CMV replication [17].

Our study population included only HIV positive MSM, and this characteristic explained the high percentage of patients with HHV-8 salivary shedding: amongst HIV-positive people, the HHV-8 seroprevalence was higher in MSM and lower in heterosexual adults with low-risk or high-risk sexual behaviour and in persons who inject drugs [32].

The most frequent virological pattern detected in the same patients at both T0 and T1 was the isolated EBV DNA shedding, with a comparable frequency in SSPs and in DV; this result is in accord with our previous data [20]. EBV persists as a latent infection of a memory B cell population, and these cells recirculate between the blood and the lymphoid tissues; lytic infection in the oropharyngeal epithelium leads to oral shedding when reactivation occurs [33], whereas CMV persists as a latent infection of the myeloid lineage, and it can reactivate to lytic infection at various sites [34]. In addition to the physio-pathological explanation, the high frequency of shedding regardless of the virological response to ART could be related to the presence of immunoactivation, which was also demonstrated in patients with successful plasma HIV RNA suppression [35]. Agudelo Hernandez et al. [36] reported that there was a trend of a positive correlation between the subclinical EBV DNA shedding rate (evaluated in different sites, included throat washings) and plasma levels of soluble CD14, which is a marker of monocyte activation, in HIV patients who have been virally suppressed on ART for a median of 7 years.

Regarding CD4+ count at nadir, we previously reported that low CD4+ cell count was the only independent variable associated with the presence of HHV-8 DNA in plasma of naïve HIV positive patients, confirming the role of immune response in controlling HHV-8 replication [37].

In contrast with the simple data of the frequency of EBV shedding, the distribution of EBV DNA load classes differed between SSPs and VPs, both in the absolute number of patients with HVL at T0 and T1 and in the frequency of subjects negative for EBV DNA at T0 who became positive with HVL at T1. Some data on the clinical significance of high EBV DNA values evaluated in saliva were published, with Kuhara et al. [38] reporting that high EBV DNA levels were observed in the onset-stage of patients with connective tissue diseases (mean value of 6.85 log_10_ EBV DNA copies/ml) and that the viral load was significantly higher than in patients in the relapse or inactive stages. Additionally, the mean EBV viral load increased significantly in kidney transplant recipients after transplantation (18,027 copies/ml before transplantation versus 192,134 copies/ml 3 months after transplantation) [39], and the EBV DNA level was higher in stage IV nasopharyngeal carcinoma patients (5.56 ± 0.31 log DNA copies/μl) than in stage I-III patients [40].

Taken together, these data suggest that the EBV DNA load may play a role in advanced cancers and that this herpesvirus can be involved in the mechanism of immune-mediated inflammation. Miller et al. [17] demonstrated that the mean EBV DNA load was higher in HIV-positive subjects with respect to healthy controls, and it increased with increasing HIV RNA values in a study involving 58 HIV positive patients. We did not include HIV-negative controls in our study because we focused on the role of long-term plasma HIV RNA control and VPs as our control population; however, we confirmed the relationship between plasma HIV RNA and EBV DNA viral load at T0. No analysis was performed at T1 because of the low number of subjects with LVL (only 3).

Isolated HHV-8 detection was identified in 2 SSPs (5%) but not in VPs, and no subjects showed isolated shedding of HHV-8 DNA at both T0 and T1. Interestingly, in 5 SSPs (12.5%) and 3 VPs (9.1%), the combined EBV-HHV-8 shedding presented at T0 was confirmed at T1. Miller et al. [17] reported that HHV-8 and EBV in saliva samples were more likely to be simultaneously present in a study population of 58 HIV-positive subjects (81% on ART) than in healthy controls; the overall percentage was 32.7%, but only one sample was tested. Carvalho et al. [18] did not include the combined EBV and HHV-8 shedding in the list of viruses most likely to be simultaneously detected, but their study population of HIV-infected subjects had an HSV-1 prevalence of 51.1%, and the combination HHV-8, HSV-1 and EBV was included instead. Gammaherpesviruses can establish a latent biological cycle in infected cells, but latently HHV-8-infected cells do not become immortalized, unlike latently EBV-infected cells; this characteristic could explain the higher frequency of the association between EBV and HHV-8 detection with respect to isolated HHV-8 positivity [41]. We reported that VPs and SSPs with HHV-8 detection had no significant differences in CD8-positive cell absolute count and percentage, unlike the patients with no shedding. We are aware that the sample size influenced the statistical significance, but coinfection with HHV-8 may be associated with an immune activation and increase of CD8/CD38/HLA-DR cells in HIV patients on effective ART [42].

Herpesvirus salivary shedding was not identified in 9 patients (9.8%, 7 SSPs and 2 PSPs); this last percentage is in accord with that reported in previous studies with a single herpesvirus salivary testing, ranging from 3 to 25% [17, 18, 20]. Conversely, we demonstrated multiple herpesvirus reactivations both at T0 and at T1 in 24 patients (26.1% of the overall study population). The significance of asymptomatic herpesvirus reactivation as a marker of clinical significance was recently studied by Ong et al. [43] in patients without known prior immunodeficiency admitted to the intensive care unit (ICU) with a diagnosis of septic shock: patients with multiple herpesvirus reactivations in plasma had a higher Intensive Care Unit mortality with respect to patients with a single-type viremia or no viremia. No definite explanation of the physiopathological mechanisms underlying these correlations is available: of note, we demonstrated the combined shedding of EBV, HHV-8 and CMV at both T0 and T1 only in VPs.

This study’s strengths include the availability of a 48-month study period composed of two 24-month intervals and the two samplings conducted at the end of each period; the inclusion of HIV-untreated patients; the description of simultaneous infections in a single patient; and the focusing on herpesviruses reactivation, with the exclusion per protocol of primary infections. The main limitation of the study is the sample size, which limits the ability to identify differences in immunovirological parameters between subgroups of patients.

## Conclusions

In conclusion, we observed that the replication of two oncogenic viruses such as EBV and HHV-8 occurred with a comparable frequency in immunocompetent patients who were either on successful ART or untreated. Moreover, the most frequent persistent multiple shedding is the combination of EBV-HHV-8. This result provides a basis for investigating the predictive role of herpesvirus salivary shedding on the development of herpesvirus-related malignancies and possibly on immunoactivation status. Saliva sampling is a non-invasive approach that can be performed via self-collection and allows for longitudinal evaluation with minimal patient effort.

## Additional file

Additional file 1: Salivary shedding of all combinations of EBV DNA, CMV DNA and HHV8-DNA shedding at T0 and at T1 in the 3 groups of HIV patients (SSP, VP, PSP). Data at T0 are expressed as absolute value and as percentage respect to the number of patients included in the specific cohort, at T1 only as absolute value. (DOCX 19 kb)

## Abbreviations

CMV: Cytomegalovirus
EBV: Epstein-Barr virus
HAART: Highly active antiretroviral therapy
HHV-8: Human herpesvirus 8
HVL: High viral load
LVL: Low viral load
MSM: Men who have sex with men
PSPs: Partially suppressed patients
SSPs: Successfully suppressed patients
VPs: Viremic patients

## References

[1] Virgin HW, Wherry EJ, Ahmed R (2009). Redefining chronic viral infection. Cell.

[2] Cruz-Muñoz ME, Fuentes-Pananá EM (2018). Beta and Gamma human herpesviruses: agonistic and antagonistic interactions with the host immune system. Front Microbiol.

[3] Gilden DH, Mahalingam R, Cohrs RJ, Tyler KL (2007). Herpesvirus infections of the nervous system. Nat Clin Pract Neurol.

[4] Sinzger C, Digel M, Jahn G (2008). Cytomegalovirus cell tropism. Curr Top Microbiol Immunol.

[5] Hau PM, Tsao SW. Epstein-Barr virus hijacks DNA damage response transducers to orchestrate its life cycle. Viruses. 2017;9. 10.3390/v9110341 PMC5707548 29144413

[6] Haidar G, Singh N (2017). Viral infections in solid organ transplant recipients: novel updates and a review of the classics. Curr Opin Infect Dis.

[7] Young LS, Yap LF, Murray PG (2016). Epstein-Barr virus: more than 50 years old and still providing surprises. Nat Rev Cancer.

[8] Dziurzynski K, Chang SM, Heimberger AB, Kalejta RF, McGregor Dallas SR, Smit M (2012). Consensus on the role of human cytomegalovirus in glioblastoma. Neuro-Oncology.

[9] Gonçalves PH, Uldrick TS, Yarchoan R (2017). HIV-associated Kaposi sarcoma and related diseases. AIDS.

[10] Uldrick TS, Wang V, O'Mahony D, Aleman K, Wyvill KM, Marshall V (2010). An interleukin-6-related systemic inflammatory syndrome in patients co-infected with Kaposi sarcoma-associated herpesvirus and HIV but without multicentric Castleman disease. Clin Infect Dis.

[11] Halenius A, Hengel H (2014). Human cytomegalovirus and autoimmune disease. Biomed Res Int.

[12] Kang I, Quan T, Nolasco H, Park SH, Hong MS, Crouch J (2004). Defective control of latent Epstein-Barr virus infection in systemic lupus erythematosus. J Immunol.

[13] Boppana SB, Ross SA, Shimamura M, Palmer AL, Ahmed A, Michaels MG (2011). Saliva polymerase-chain-reaction assay for cytomegalovirus screening in newborns. N Engl J Med.

[14] Pow EH, Law MY, Tsang PC, Perera RA, Kwong DL (2011). Salivary Epstein-Barr virus DNA level in patients with nasopharyngeal carcinoma following radiotherapy. Oral Oncol.

[15] Parisi SG, Cruciani M, Scaggiante R, Boldrin C, Andreis S, Dal Bello F (2011). Anal and oral human papillomavirus (HPV) infection in HIV-infected subjects in northern Italy: a longitudinal cohort study among men who have sex with men. BMC Infect Dis.

[16] Dittmer DP, Tamburro K, Chen H, Lee A, Sanders MK, Wade TA (2017). Oral shedding of herpesviruses in HIV-infected patients with varying degrees of immune status. AIDS.

[17] Miller CS, Berger JR, Mootoor Y, Avdiushko SA, Zhu H, Kryscio RJ (2006). High prevalence of multiple human herpesviruses in saliva from human immunodeficiency virus-infected persons in the era of highly active antiretroviral therapy. J Clin Microbiol.

[18] Carvalho KS, Silvestre Ede A, Maciel Sda S, Lira HI, Galvão RA, Soares MJ (2010). PCR detection of multiple human herpesvirus DNA in saliva from HIV-infected individuals in Teresina, state of Piauí, Brazil. Rev Soc Bras Med Trop.

[19] Jacobson MA, Ditmer DP, Sinclair E, Martin JN, Deeks SG, Hunt P (2009). Human herpesvirus replication and abnormal CD8+ T cell activation and low CD4+ T cell counts in antiretroviral-suppressed HIV-infected patients. PLoS One.

[20] Scaggiante R, Andreis S, Basso M, Franchin E, Franzetti M, Del Vecchio C (2016). Epstein-Barr and cytomegalovirus DNA salivary shedding correlate with long-term plasma HIV RNA detection in HIV-infected men who have sex with men. J Med Virol.

[21] May MT, Gompels M, Delpech V, Porter K, Orkin C, Kegg S (2014). Impact on life expectancy of HIV-1 positive individuals of CD4+ cell count and viral load response to antiretroviral therapy. AIDS.

[22] Hofstra LM, Mudrikova T, Stam AJ, Otto S, Tesselaar K, Nijhuis M (2014). Residual viremia is preceding viral blips and persistent low-level viremia in treated HIV-1 patients. PLoS One.

[23] Young J, Rickenbach M, Calmy A, Bernasconi E, Staehelin C, Schmid P (2015). Transient detectable viremia and the risk of viral rebound in patients from the Swiss HIV cohort study. BMC Infect Dis.

[24] Navazesh M (1993). Methods for collecting saliva. Ann N Y Acad Sci.

[25] Parisi SG, Basso M, Del Vecchio C, Andreis S, Franchin E, Bello FD (2016). Virological testing of cerebrospinal fluid in children aged less than 14 years with a suspected central nervous system infection: a retrospective study on 304 consecutive children from January 2012 to May 2015. Eur J Paediatr Neurol.

[26] Biasolo MA, Calistri A, Cesaro S, Gentile G, Mengoli C, Palu G (2003). Case report: kinetics of Epstein-Barr virus load in a bone marrow transplant patient with no sign of lymphoproliferative disease. J Med Virol.

[27] Mengoli C, Cusinato R, Biasolo MA, Cesaro S, Parolin C, Palu G (2004). Assessment of CMV load in solid organ transplant recipients by pp65 antigenemia and real-time quantitative DNA PCR assay: correlation with pp67 RNA detection. J Med Virol.

[28] White IE, Campbell TB (2000). Quantitation of cell-free and cell-associated Kaposi’s sarcoma-associated herpesvirus DNA by real-time PCR. J Clin Microb.

[29] Conover WJ. Practical nonparametric statistics. 3rd ed. New York: John Wiley & Sons.

[30] European AIDS Clinical Society Guidelines, Version 7.1. http://www.eacsociety.org. Accessed 12 Oct 2017.

[31] European AIDS Clinical Society Guidelines, Version 9.0. http://www.eacsociety.org. Accessed 18 Jan 2018.

[32] Rohner E, Wyss N, Heg Z, Faralli Z, Mbulaiteye SM, Novak U (2016). HIV and human herpesvirus 8 co-infection across the globe: systematic review and meta-analysis. Int J Cancer.

[33] Hislop AD, Taylor GS, Sauce D, Rickinson AB (2007). Cellular responses to viral infection in humans: lessons from Epstein-Barr virus. Annu Rev Immunol.

[34] Crough T, Khanna R (2009). Immunobiology of human cytomegalovirus: from bench to bedside. Clin Microbiol Rev.

[35] Hattab S, Guiguet M, Carcelain G, Fourati S, Guihot A, Autran B (2015). Soluble biomarkers of immune activation and inflammation in HIV infection: impact of 2 years of effective first-line combination antiretroviral therapy. HIV Med.

[36] Agudelo-Hernandez A, Chen Y, Bullotta A, Buchanan WG, Klamar-Blain CR, Borowski L (2017). Subclinical herpesvirus shedding among HIV-1-infected men on antiretroviral therapy. AIDS.

[37] Parisi SG, Boldrin C, Andreis S, Ferretto R, Fuser R, Malena M (2011). KSHV DNA viremia correlates with low CD4+ cell count in Italian males at the time of diagnosis of HIV infection. J Med Virol.

[38] Kuhara T, Watanabe D, Ishida N, Tamada Y, Matsumoto Y, Ihira M (2013). Quantitative analysis of shedding of Epstein-Barr virus in saliva from patients with connective tissue diseases: a pilot study. Int J Dermatol.

[39] Nikoobakht M, Beitollahi J, Nikoobakht N, Aloosh M, Sahebjamee M, Rezaeidanesh M (2011). Evaluation of Epstein-Barr virus load in saliva before and after renal transplantation. Transplant Proc.

[40] Shan J, Pow EH, Tsang PC, Perera RA, Kwong DL (2014). Comparison of two laboratory extraction techniques for the detection of Epstein-Barr virus in the saliva of nasopharyngeal carcinoma patients. J Investig Clin Dent.

[41] Mui UN, Haley CT, Tyring SK. Viral oncology: molecular biology and pathogenesis. J Clin Med. 2017;6. 10.3390/jcm6120111 PMC5742800 29186062

[42] Masiá M, Robledano C, Ortiz de la Tabla V, Antequera P, Lumbreras B, Hernández I (2014). Coinfection with human herpesvirus 8 is associated with persistent inflammation and immune activation in virologically suppressed HIV-infected patients. PLoS One.

[43] Ong DSY, Bonten MJM, Spitoni C, Verduyn Lunel FM, Frencken JF, Horn J (2017). Epidemiology of multiple herpes viremia in previously immunocompetent patients with septic shock. Clin Infect Dis.

