# Supplementary material for: Cytomegalovirus, Epstein-Barr virus and human herpesvirus 8 salivary shedding in HIV positive men who have sex with men with controlled and uncontrolled plasma HIV viremia: a 24-month longitudinal study
Source: BMC Infect Dis. 2018 Dec 19;18:683. doi: 10.1186/s12879-018-3591-x (PMC6300014; doi:10.1186/s12879-018-3591-x)
Supplement: Supplementary file 1 — Salivary shedding of all combinations of EBV DNA, CMV DNA and HHV8-DNA shedding at T0 and at T1 in the 3 groups of HIV patients (SSP, VP, PSP). Data at T0 are expressed as absolute value and as percentage respect to the number of patients included in the specific cohort, at T1 only as absolute value. (DOCX 19 kb) [file 12879_2018_3591_MOESM1_ESM.docx]

**Additional file 1** Salivary shedding of all combinations of EBV DNA, CMV DNA and HHV8-DNA shedding at T0 and at T1 in the 3 groups of HIV patients (SSP, VP, PSP). Data at T0 are expressed as absolute value and as percentage respect to the number of patients included in the specific cohort, at T1 only as absolute value.

a) HIV Successfully suppressed patients (n=40)

| **T0** | **T1** | | | | | | | |
| --- | --- | --- | --- | --- | --- | --- | --- | --- |
|  | No  shedding | Isolated  CMV shedding | Isolated  EBV  shedding | Isolated  HHV-8  shedding | CMV+EBV  shedding | CMV+HHV-8 shedding | EBV+HHV-8  shedding | CMV+EBV+HHV-8 shedding |
| No shedding  11 pts (27.5%) | 7 pts | - | 2 pts | 1 pt | - | - | 1 pt | - |
| Isolated CMV shedding  0 pts | - | - | - | - | - | - | - | - |
| Isolated EBV shedding  13 pts (32.5%) | - | - | 6 pts | 1 pt | 5 pts | - | 1 pt | - |
| Isolated HHV-8 shedding  2 pts (5%) | 1 pt | - | - | - | - | - | 1 pt | - |
| CMV+EBV shedding  2 pts (5%) | - | 1 pt | 1 pt | - | - | - | - | - |
| CMV+HHV-8  shedding  0 pts | - | - | - | - | - | - | - | - |
| EBV+HHV-8  shedding  9 pts (22.5%) | 1 pt | - | 3 pts | - | - | - | 5 pts | - |
| CMV+EBV  +HHV-8 shedding  3 pts (7.5%) | - | - | - | 1 pt | - | - | 2 pts | - |
|  |  |  |  |  |  |  |  |  |

b) HIV viremic patients (n=33)

| **T0** | **T1** | | | | | | | |
| --- | --- | --- | --- | --- | --- | --- | --- | --- |
|  | No  shedding | Isolated  CMV shedding | Isolated  EBV shedding | Isolated  HHV-8 shedding | CMV+EBV  shedding | CMV+HHV-8 shedding | EBV+HHV-8  shedding | CMV+EBV+HHV-8 shedding |
| No shedding  4 pts (12.1%) | - | 1 pt | 1 pt | - | 1 pt | - | 1 pt | - |
| Isolated CMV shedding  0 pts | - | - | - | - | - | - | - | - |
| Isolated EBV  shedding  11 pts (33.3%) | 1 pt | - | 7 pts | - | 1 pt | - | 2 pts | - |
| Isolated HHV-8  shedding  0 pts | - | - | - | - | - | - | - | - |
| CMV+EBV  shedding  3 pts (9.1%) | - | - | - | - | - | - | 1 pt | 2 pts |
| CMV+HHV-8  shedding  1 pts (3%) | - | - | - | - | 1 pt | - | - | - |
| EBV+HHV-8  shedding  10 pts (30.3%) | 2 pts | 1 pt | 1 pt | 1 pt | 1 pt | - | 3 pts | 1 pt |
| CMV+EBV+HHV-8  shedding  4 pts (12.1%) | - | - | - | - | 1 pt | - | 1 pt | 2 pts |

c) HIV partially suppressed patients (n=19)

| **T0** | **T1** | | | | | | | |
| --- | --- | --- | --- | --- | --- | --- | --- | --- |
|  | No  shedding | Isolated  CMV shedding | Isolated  EBV shedding | Isolated  HHV-8 shedding | CMV+EBV  shedding | CMV+HHV-8  shedding | EBV+HHV-8  shedding | CMV+EBV+HHV-8 shedding |
| No shedding  3 pts (15.8%) | 2 pts | - | 1 pt | - | - | - | - | - |
| Isolated CMV shedding  1 pt (5.3%) | 1 pt | - | - | - | - | - | - | - |
| Isolated EBV shedding  7 pts (36.8%) | 1 pt | - | 5 pts | - | 1 pt | - | - | - |
| Isolated HHV-8 shedding  1 pts (5.3%) | - | - | - | - | - | - | 1 pt | - |
| CMV+EBV shedding  2 pts (10.5%) | 1 pt | - | - | - | 1 pt | - | - | - |
| CMV+HHV-8 shedding  1 pts (5.3%) | - | - | 1 pt | - | - | - | - | - |
| EBV+HHV-8 ahedding  3 pts (15.8%) | - | - | - | - | - | - | 3 pts | - |
| CMV+EBV+HHV-8  shedding  1 pts (5.3%) | - | - | 1 pt | - | - | - | - | - |
